# *Rickettsia massiliae* Human Isolation

**DOI:** 10.3201/eid1201.050850

**Published:** 2006-01

**Authors:** Giustina Vitale, Serafino Mansueto, Jean-Marc Rolain, Didier Raoult

**Affiliations:** * Azienda Ospedaliera Universitaria Policlinico "P. Giaccone," Palermo, Italy;; † Université de la Méditerranée, Marseille, France

**Keywords:** Rickettsia massiliae, first isolation, blood patient

**To the Editor:** The number of new rickettsial species that cause diseases in humans is rapidly increasing (1). Moreover, many of the species first described in ticks have been recently shown to be pathogenic. Of the 10 species or subspecies found to be pathogens after 1984, a total of 7 were first isolated from ticks (2). We report the first isolation of *Rickettsia massiliae* from a patient. The bacterium was isolated in Sicily in 1985 and identified in 2005.

A 45-year-old man was hospitalized in Palermo, Italy, on June 6, 1985, for fever and a rash. He had been febrile since May 25 and did not respond to antimicrobial drug treatment using cefamezin, a first-generation cephalosporin. On examination, he had a necrotic eschar on his right ankle, a maculopapular rash on his palms and soles (Figure A1), and slight hepatomegaly. Leukocyte count was normal; he received tetracyclines for 13 days and fully recovered. He seroconverted (from 0 to 1:80 between day 11 and day 24) by indirect immunofluorescence to *Rickettsia conorii* (*R. conorii* spot, bioMérieux, Marcy l'Étoile, France).

Four milliliters of heparinized blood sampled before treatment were inoculated in a 25-cm^2^ flask containing Vero cells and incubated at 33°C in a CO_2_ incubator (1). Direct immunofluorescence test on a sample of the patient's serum was positive 7 days later. The strain was stored for 20 years and tested in 2005 at the Unité des Rickettsies for identification, and *R. massiliae* was identified. DNA was extracted from the cell culture supernatant and used as template in 2 previously described polymerase chain reaction (PCR) assays that targeted a portion of the rickettsial *ompA* gene as well as a portion of the rickettsial *gltA* gene (3,4). Amplification products of the expected size were obtained from this extract but from no concurrently processed control materials, including 3 negative controls. DNA sequencing of the positive PCR products gave 100% identity with *R. massiliae* for *ompA* (GenBank accession no. RBU43792) and 99.9% homology for *gltA* (GenBank accession no. RSU 59720).

*R. massiliae* was first isolated from *Rhipicephalus* ticks in Marseilles (5). It is transmitted transovarially in *Rhipicephalus turanicus* (2). *R. massiliae* is commonly found in *Rhipicephalus sanguineus* or *R. turanicus* in France, Greece, Spain (identified as Bar 29) (6), Portugal, Switzerland, Sicily (D. Raoult, unpub. data), Central Africa, and Mali (2). *R. massiliae* may be commonly associated with these ticks, which are distributed worldwide.

*R. massiliae* is grouped phylogenically with *Rickettsia rhipicephali* and *Rickettsia aeschlimannii* (Figure A2). Bacteria from this group have a natural resistance to rifampin that is associated with an *rpo*B sequence that is different from that of other rickettsiae. This isolate was not tested for antimicrobial drug susceptibly (7). Rifampin resistance leads us to believe that this isolate may cause a Mediterranean spotted fever–like disease that was described in children in Spain (7,8). Serologic findings were recently reported that showed some patients in Barcelona, Spain, with reactions that indicate *R. massiliae* (B29 strain) rather than *R. conorii* (6). However, serologic reactions are only presumptive; isolation from a patient is the required to initially describe a new disease (9).

This Sicilian index case shows that *R. massiliae* is a human pathogen. It contraindicates using rifampin to treat Mediterranean spotted fever in areas where *R. massiliae* is endemic, as it cannot as yet be differentiated from *R. conorii* infection. *R. massiliae* is a new example of a strain identified in ticks for several years before its first isolation from a human patient (10). The longest delay was observed for *Rickettsia parkeri*, which was isolated from ticks in 1939 but not from a patient until 2004. Many authors labeled *R. parkeri* a nonpathogenic rickettsia during this time (1). In the present case, the human isolate was obtained before the tick isolate but was not further identified. When this strain was isolated, *R. conorii* was the sole *Rickettsia* sp. found in ticks in southern Europe. Moreover, only 1 tickborne pathogenic *Rickettsia* sp. was believed to circulate in a single area. Since that time, several tickborne rickettsial diseases have been shown to exist in the same area, which prompted us to retrospectively identify this strain. The patient was reexamined in May 2005, after this identification. He is healthy and has no remaining antibodies against *Rickettsia* spp.

## Figures and Tables

**Figure A1:**
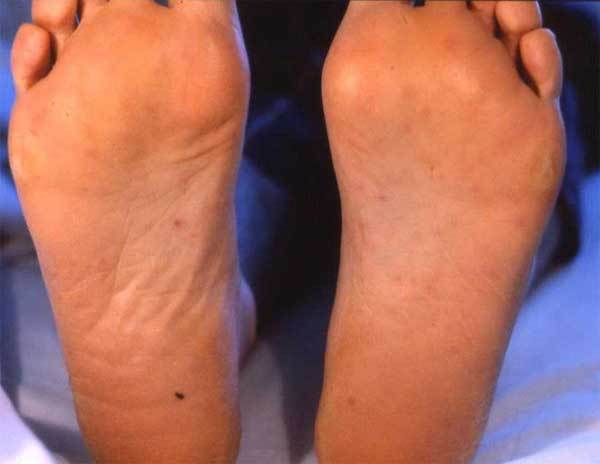
Maculopapular rash on the soles of the patient's feet.

**Figure A2:**
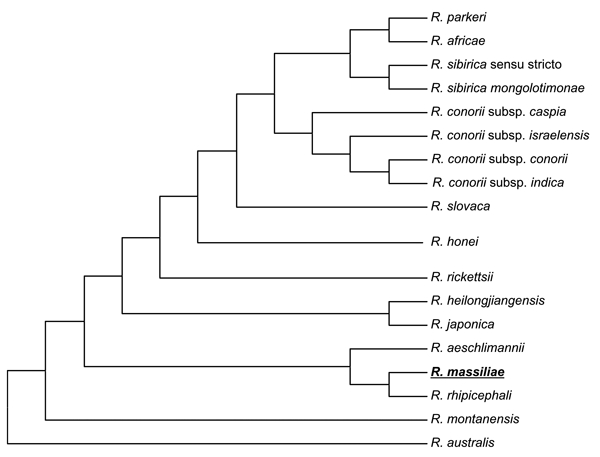
Phylogenetic tree of rickettsiae including *Rickettsia massiliae* obtained by comparing partial sequences of *ompA* with the parsimony method.
